# Timing influences detection of anti-nephrin antibodies in recurrent FSGS. Reply.

**DOI:** 10.1172/JCI213243

**Published:** 2026-11-16

**Authors:** Luca Perico, Federica Casiraghi, Ariela Benigni, Giuseppe Remuzzi

**Affiliations:** Istituto di Ricerche Farmacologiche Mario Negri IRCCS, Bergamo, Italy.

**Keywords:** Immunology, Nephrology, Adaptive immunity, Antigen, Autoimmune diseases

**The authors reply:** We thank Lafargue and Riella for their interest in our work (1). Our study was designed to determine whether anti-nephrin antibodies have a pathogenetic role in posttransplant focal segmental glomerulosclerosis (FSGS) recurrence or can only be used as a marker for disease activity to drive therapeutic decisions (2). The statement that “the timing of recurrence appears atypical for classical recurrent FSGS” does not apply to our cohort. As noted by Lafargue and Riella, the mean time from transplantation to recurrence was 569 days in the 4 adults sampled during recurrence. However, this was largely driven by 2 patients with atypically late recurrences. This pattern was not representative of the cohort as a whole, as the remaining patients recurred within days to weeks, reflecting the timing indicated by the authors (1). Notably, all 3 pediatric patients sampled during recurrence that developed within 2–15 days after transplantation, exactly within the classical early-recurrence window, were antibody negative (2). Therefore, the timing of recurrence in our cohort cannot be described as “often late” as indicated in Table 1, but as “predominantly early,” as in the TANGO consortium.

Among 34 patients with posttransplant recurrence, 5 were sampled before transplantation and all were negative (2). The same applies to 7 patients (4 adults and 3 pediatric patients) sampled during active recurrence. Possible treatment effects were not an issue, since all samples were obtained within 10 days of diagnosis and before plasmapheresis or rituximab treatment. More importantly, allograft biopsies (*n* = 6) obtained at recurrence showed neither glomerular IgG deposition nor IgG-nephrin colocalization by either confocal microscopy or super-resolution microscopy.

In their letter, Lafargue and Riella describe TANGO as providing a contrasting perspective to ours (1). However, we found our cohort and theirs were strongly consistent. Specifically, the preliminary analysis of the TANGO study presented at the 2025 American Society of Nephrology annual meeting included 161 kidney transplant recipients with end-stage kidney disease attributed to FSGS and identified only 7 anti-nephrin–positive patients (4.3%) (3). Seropositivity was observed in 5/30 patients with recurrence versus 2/45 without recurrence, with no statistically significant association. In the current letter, they report an analysis from the same consortium in which 136 patients were included, of whom 11 were positive, with a significant association with recurrence (73% vs. 38%; HR 2.62) (1). It is important to clarify why, despite a smaller cohort presumably derived from the same population, the proportion of positive samples increased modestly (8%). In any case, the 2 analyses converge: anti-nephrin positivity in FSGS recurrence is extremely uncommon, as our study concluded.

We are therefore not convinced that their data and ours support a more nuanced role of these antibodies in a specific clinical context during recurrent FSGS. We offer an alternative interpretation based on a number of facts. First, a recent study by Raglianti et al. showed that isolated anti-nephrin positivity was not associated with recurrent FSGS (4). In fact, the presence of anti-nephrin antibodies was associated with preserved kidney function, sustained remission, and lower likelihood of FSGS. This is difficult to reconcile with a simple model in which anti-nephrin antibodies alone confer a more severe or treatment-resistant phenotype. Second, anti-nephrin antibodies have been reported at a similar prevalence (8%) in patients with IgA nephropathy, stressing again the possibility of a nonspecific epiphenomenon of podocyte stress and heavy proteinuria (5).

How do we reconcile all of this? In our view, podocyte-directed autoreactivity may arise from podocyte damage following an initial injury, through increased availability of cryptic podocyte antigens (4). The best example of this phenomenon in human disease is Dressler syndrome (6). This syndrome develops when cardiac tissue injury, resulting from myocardial infarction, cardiac surgery, or other myocardial or pericardial damage, exposes cardiac antigens and triggers an immune response with development of a variety of anti-myocardial antibodies and immune complex–mediated inflammation (6).

Finally, in their letter, Lafargue and Riella (1) attempt to establish anti-nephrin antibodies as a clinically actionable marker of a distinct subset of posttransplant recurrent patients, with prognostic value and potential to guide immunosuppression, ideally B cell–targeted therapies. However, this interpretation is difficult to reconcile with the broad efficacy of B cell depletion across podocytopathies. In adults and children, rituximab can induce or maintain remission, reduce immunosuppression requirements in steroid-dependent or frequently relapsing nephrotic syndrome, and halt disease-associated growth impairment in children (7).

## Version 1 11/16/2026

Electronic publication
